# Inpatient Personality Disorder Presentations in Qatar: A First Epidemiological and Service Audit

**DOI:** 10.1192/bjo.2026.11561

**Published:** 2026-06-30

**Authors:** Sazgar Hamad, Aisha Alkhulaifi, Sedakat Dinc, Maryam Almalki, AHMAD ALATER

**Affiliations:** Hamad Medical Corporation, Doha, Qatar

## Abstract

**Aims:**

In Qatar, there are currently no published local data and no standardised inpatient policy/care pathway for personality disorder (PD) management. This creates variability in assessment, crisis planning, psychological input, and prescribing practice. We aimed to establish a baseline epidemiological profile of inpatient PD presentations and describe current inpatient management processes to inform service development. We hypothesised that borderline personality disorder (BPD) would be the most frequent PD diagnosis and that care processes would show wide variability in the absence of a local guideline.

**Methods:**

We conducted a retrospective audit within Hamad Medical Corporation Mental Health Services using electronic health record review (Cerner) and structured case-note abstraction. All eligible psychiatric admissions between January and December 2025 were included (N=171). Extracted variables included PD diagnosis and type, documentation supporting BPD diagnostic criteria, MDT review, crisis planning, documented individual and group psychotherapy sessions, inpatient self-harm incidents, documented substance use, and psychotropic prescribing.

**Results:**

A personality disorder diagnosis was recorded in 83 of 171 admissions (48.5%); the remaining 88 admissions (51.5%) did not carry a documented personality disorder diagnosis during the admission period. BPD was the most frequent PD diagnosis (54/83, 65.1%), followed by antisocial PD (13/83, 15.7%). Mean age was 26.8 years (SD 10.9); 55/83 (66.3%) were female and 49/83 (59.0%) were readmissions. For BPD admissions, documentation supported ≥5 diagnostic criteria in 40/54 (74.1%) (median 6 criteria). MDT review was documented in 72/83 (86.7%) and a crisis plan in 51/83 (61.4%). Individual psychotherapy session counts were recorded for 48/83 (57.8%); of these, 26/48 (54.2%) had zero documented sessions. Group psychotherapy session counts were recorded for 51/83(61.4%); of these, 34/51 (66.7%) had zero documented sessions. Self-harm incidents occurred in 21/83 (25.3%), and substance use was documented in 33/83 (39.8%). Antipsychotics were prescribed in 58/83 (69.9%) and benzodiazepines in 33/83 (39.8%).

**Conclusion:**

This first inpatient PD audit in Qatar shows a substantial service burden dominated by BPD, high readmission rates, limited documented psychological input, and incomplete crisis planning. In the context of no local policy/guideline, the findings support developing a standardised inpatient PD pathway by adopting international guidance (with cultural adaptation for the region), strengthening crisis planning and psychological interventions, and reviewing prescribing practice–particularly reducing benzodiazepine use and promoting safer alternatives.

